# *Yersinia pseudotuberculosis* growth arrest during type-III secretion system expression is associated with altered ribosomal protein expression and decreased gentamicin susceptibility

**DOI:** 10.1371/journal.ppat.1012548

**Published:** 2025-07-07

**Authors:** Justin Greene, Katherine L. Cotten, Rhett A. Snyder, Ryan C. Huiszoon, Sangwook Chu, Rezia Era D. Braza, Ashley A. Chapin, Justin M. Stine, William E. Bentley, Reza Ghodssi, Kimberly M. Davis

**Affiliations:** 1 W. Harry Feinstone Department of Molecular Microbiology and Immunology, Johns Hopkins Bloomberg School of Public Health, Baltimore, Maryland, United States of America; 2 Institute for Systems Research, University of Maryland, College Park, Maryland, United States of America; 3 Fischell Department of Bioengineering, University of Maryland, College Park, Maryland, United States of America; 4 Robert E. Fischell Institute for Biomedical Devices, University of Maryland, College Park, Maryland, United States of America; 5 Department of Electrical and Computer Engineering, University of Maryland, College Park, Maryland, United States of America; University of Virginia School of Medicine, UNITED STATES OF AMERICA

## Abstract

It has been long appreciated that expression of the *Yersinia* type-III secretion system (T3SS) in culture is associated with growth arrest. Here we sought to understand whether T3SS expression is sufficient to trigger loss of exponential phase markers, and utilized a fluorescent reporter for ribosomal protein expression to detect changes in bacterial growth state. Using a fluorescent transcriptional reporter with the *rpsJ*/S10 promoter fused to a destabilized *gfp* variant, we confirmed reporter expression significantly increases in exponential phase and decreases as cells transition to stationary phase. In a mouse model of systemic *Y. pseudotuberculosis* infection, we found multiple subsets of bacterial cells in the mouse spleen, including cells with high T3SS and low S10 expression and cells with high expression of both markers. In bacterial media, growth inhibition with T3SS induction and a reduction in S10 expression were observed, but a significant proportion of cells retained high expression of both T3SS and S10. Paradoxically, while loss of T3SS expression rescued growth, lower S10 expression was detected, again indicating bacteria can express both markers simultaneously. In media, bacteria grow planktonically as individual cells, while in mouse tissues, bacteria form clustered extracellular communities. We utilized droplet-based microfluidics to encapsulate bacteria in spherical agarose droplets and model clustered growth, and observed high expression of T3SS without an impact on S10 levels. Finally, we show that T3SS expression is sufficient to promote antibiotic tolerance, but surviving bacteria in a gentamicin treatment mouse model specifically express low S10. Collectively, these data indicate that the growth arrest associated with T3SS induction can reduce antibiotic susceptibility, but cells surviving antibiotic treatment display lower levels of the exponential phase marker, S10.

## Author summary

Slow-growing bacterial cells generally have reduced antibiotic susceptibility, rendering them difficult to eliminate during antibiotic treatment. However, for many key virulence factors (bacterial factors required to promote infection), it remains unclear whether expression is sufficient to slow bacterial growth and impact antibiotic susceptibility. Expression of one of the critical virulence factors for *Yersinia,* the type-III secretion system (T3SS), has long been known to slow or arrest bacterial growth, but the impact of T3SS expression on antibiotic susceptibility had not yet been explored. Using fluorescent reporter-based strategies, and combinations of culture-based experiments and mouse models, we show that high levels of T3SS expression can promote slowed growth and antibiotic tolerance. However, we also identify subsets of T3SS-expressing cells that maintain markers of exponential phase growth, and we show here that the level of exponential phase markers dictates the antibiotic susceptibility of individual cells.

## Introduction

Exposure to host-derived antimicrobials and immune cell interactions trigger expression of bacterial virulence factors, components required to combat host defenses and promote disease. While virulence factors are necessary during infection, high levels of expression can alter bacterial cell physiology, and impact overall fitness to the point of promoting slowed growth and reduced antibiotic susceptibility [1]. Further, virulence factor expression is often heterogeneous across a bacterial population which may promote differential antibiotic susceptibility amongst individual cells [1–3]. Mixed populations of rapidly-growing, antibiotic-susceptible cells and slowly-growing, antibiotic-tolerant cells are termed antibiotic persistent populations [4,5]. It can be very difficult to fully eliminate these populations during antibiotic treatment, resulting in residual subpopulations of bacterial cells capable of causing relapsing infection. It has also been shown that persistent populations can give rise to antibiotic resistant cells [6,7], linked to prolonged exposure of persistent cells to host-derived stressors and antibiotic stress during courses of antibiotic treatment. For these reasons, it is critical to better understand the mechanisms underlying alterations in antibiotic susceptibility and explore treatment regiments which are more effective at eliminating the entirety of bacterial populations.

One of the central virulence factors for Gram-negative bacterial pathogens is a type-III secretion system (T3SS), which injects its associated effector proteins into the host cell cytoplasm to modulate host cell processes [8]. For human pathogenic *Yersiniae* (*Yersinia pseudotuberculosis, Yersinia enterocolitica, Yersinia pestis*), the T3SS effectors uncouple host signaling cascades, limit reactive oxygen species production, and prevent phagocytosis to allow bacteria to remain in an extracellular niche [9–13]. Expression of the *Yersinia* T3SS has been shown to arrest growth in culture [14,15]; however, it remains unclear if expression is linked to slowed growth in mouse models of infection, or if T3SS expression is sufficient to reduce antibiotic susceptibility. These questions have been difficult to assess with bacterial genetic approaches, because of limited host immune cell infiltration in the absence of T3SS [16,17], which simultaneously removes potential fitness costs and selective pressures. Moreover, the link between T3SS expression and antibiotic susceptibility has only been explored in the context of *Salmonella enterica* serovar Typhimurium (*S.* Typhimurium) SPI-1, a T3SS which, in contrast with *Yersinia*, promotes bacterial invasion [18]. Thus, it remains unclear if expression of T3SSs more broadly impact the antibiotic susceptibility of Gram-negative pathogens.

*Y. pseudotuberculosis* is a natural pathogen of rodents and humans, and has been used since the 1960’s to study different aspects of host-pathogen interactions, such as host cell invasion [19], dynamics of virulence factor expression [20–22], dissemination and bacterial population bottlenecks [23–25], generation of immune memory in gastrointestinal-associated lymphoid tissues [26,27], inflammasome activation [16,17], and several different aspects of T3SS biology, including structure determination [28,29], host cell targeting [30,31], and regulation of expression [22,32,33]. Knowledge gained from these studies has provided critical insight into host-pathogen interactions. In the mouse model of *Y. pseudotuberculosis* systemic infection, we have previously shown that extracellular bacteria replicate to form clonal clusters, called microcolonies, in the spleen [20]. Bacteria at the periphery of microcolonies are in direct contact with neutrophils and express very high levels of the T3SS [20]. Bacteria at the center of microcolonies lack host cell contact and have an intermediate level of T3SS expression, which is induced at mammalian body temperatures (37^o^ C) [22,34]. In addition to expressing high levels of T3SS, bacteria at the periphery of microcolonies are also exposed to other host-derived stressors, such as high levels of nitric oxide (NO), which is sufficient to slow the growth of bacteria and impact antibiotic susceptibility. In previous studies, we identified peripheral cells exposed to NO and showed they exhibited slowed growth and decreased antibiotic susceptibility [3,21,35]. However, it remained unclear whether heightened T3SS expression contributed to these phenotypes alongside NO exposure.

Here, we first characterized the transcriptional signature of exponential phase *Y. pseudotuberculosis* and detected high levels of ribosomal protein transcripts in rapidly growing cells. We then generated a transcriptional reporter by fusing the promoter upstream of the ribosomal protein operon *rpsJ*/S10 to destabilized *gfp* and showed that rapidly growing cells have heightened S10 reporter expression. Within the mouse spleen, multiple subpopulations of bacteria were present, including bacteria low T3SS expression and higher S10, and cells that expressed both markers. We then used two systems to model differential T3SS expression in culture: planktonic growth in bacteriological media and an agarose droplet system, which models clustered bacterial growth. We show that high levels of T3SS expression are sufficient to slow bacterial growth, and reduce susceptibility to the ribosome-targeting antibiotic, gentamicin. However, S10 expression levels were a better predictor of bacterial survival, and in culture and in the mouse spleen, bacteria expressing high levels of S10 were eliminated by gentamicin.

## Results

### Exponential phase cells express high levels of ribosomal protein genes

**Fig 1:**
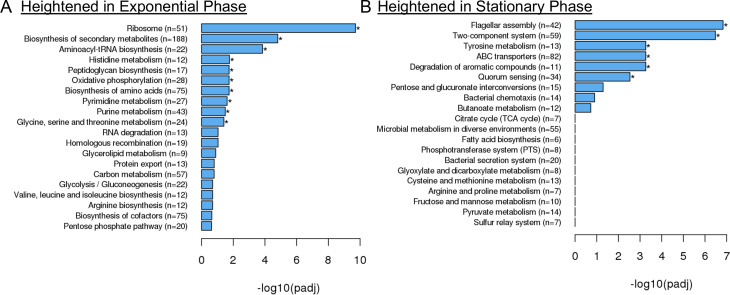
Exponential phase cells express high levels of ribosomal protein genes. RNA-seq was performed on exponential and stationary phase WT *Y. pseudotuberculosis* cells. The DESeq2 method of pairwise comparisons was used to determine significant differences in transcript levels. KEGG pathway analyses were utilized to determine the biological pathways overrepresented in **(A)** exponential and **(B)** stationary phase cells.

To determine whether T3SS expression is sufficient to stall bacterial growth to the point that cells lose exponential phase markers, we sought to generate a fluorescent reporter that would differentiate between rapidly-growing exponential and slower-growing stationary phase cells. To identify candidate promoter regions for an exponential phase transcriptional reporter, we utilized RNA-seq to determine the transcriptional profile of exponential phase *Yersinia pseudotuberculosis* as compared to stationary phase cells. Wild-type (WT) *Y. pseudotuberculosis* cells were cultured overnight (stationary phase) and subcultured in fresh media (exponential phase) at 26^o^ C to avoid spontaneous virulence plasmid loss, which can occur at a relatively high frequency during prolonged culture at 37^o^ C [36–38]. We found 894 transcripts increased more than 2-fold in exponential phase, 806 genes increased more than 2-fold in stationary phase, and 2255 protein coding genes were unchanged (S1 and S2 Tables). KEGG pathway analyses indicated that transcripts for genes associated with the ribosome, biosynthesis of secondary metabolites, and aminoacyl-tRNA biosynthesis were heightened in exponential phase (Fig 1A), while transcripts for flagellar assembly and two-component systems were heightened in stationary phase (Fig 1B). Additional groups of genes expressed in stationary phase included genes involved in tyrosine metabolism, ABC transporters, degradation of aromatic compounds, and quorum sensing (Fig 1B). Enrichment of these KEGG pathways was somewhat expected, based on RNA-seq analyses in other *Y. pseudotuberculosis* strains [39]. Of note, we found transcript levels of 56 genes were greater than 10-fold more abundant in exponential compared to stationary phase, and 22 of these 56 genes were ribosomal protein genes (Table 1). This set included *rpsJ*, which encodes for the 30S ribosomal protein S10, and whose transcripts were ~13 fold more abundant in exponential phase. *rpsJ* is the first gene expressed in a large operon of small and large ribosomal subunit protein genes (S1 Fig), suggesting that activity of the promoter upstream of *rpsJ* could serve as a reporter for transcription of the operon. Consistent with this assumption, all genes in this operon (encoding for: S10, L3, L4, L23, L2, S19, L22, S3, L16, L29, S17) were among the top 56 most highly upregulated genes in exponential phase, with more than 10-fold increased transcript abundance in exponential phase cells (S1 Fig, Table 1). Additionally, *rpsJ*/S10 expression is transcriptionally regulated by DksA and ppGpp in stationary phase, indicating a reporter containing the *rpsJ* leader and promoter sequences could reflect DksA and ppGpp-dependent transcriptional regulation of other ribosomal genes as well [40]. For these reasons, we chose the leader and promoter sequences upstream of the S10 ribosomal protein gene (*rpsJ*) for our ribosomal reporter construct to mark rapidly growing cells.

**Table 1:** Genes with highest fold change in exponential compared to stationary phase. Genes with >10 fold enrichment are shown, ribosomal genes are highlighted with bolded text. *rpsJ*/S10 is highlighted with a black box.

log2foldchange	Fold change	padjust	gene_name	gene_description
5.696331523	51.8521363	1.34E-128	ompC	porin OmpC
5.352923512	40.8686735	4.86E-44	DN756_RS01950	hypothetical protein
5.223072614	37.3509392	4.35E-44	DN756_RS19535	heme anaerobic degradation radical SAM methyltransferase ChuW/HutW
**4.90850355**	**30.0335592**	**1.46E-46**	**rplD**	**50S ribosomal protein L4**
**4.842504065**	**28.6905569**	**9.16E-50**	**rplW**	**50S ribosomal protein L23**
4.747050226	26.8537233	1.68E-54	rnpB	RNase P RNA component class A
**4.746679269**	**26.8468193**	**3.84E-54**	**rplB**	**50S ribosomal protein L2**
4.699349382	25.9803576	1.33E-28	hutX	heme utilization cystosolic carrier protein HutX
**4.624182865**	**24.6614012**	**1.22E-67**	**rplV**	**50S ribosomal protein L22**
4.587681967	24.0452824	3.91E-18	DN756_RS14930	tRNA-Gln
**4.508151871**	**22.7556339**	**5.79E-49**	**rpsS**	**30S ribosomal protein S19**
4.478526293	22.2931147	1.06E-18	DN756_RS14935	tRNA-Gln
4.455238008	21.9361435	1.66E-79	ilvC	ketol-acid reductoisomerase
**4.435700792**	**21.641083**	**6.14E-31**	**tsf**	**elongation factor Ts**
**4.402357007**	**21.1466468**	**1.53E-35**	**rplC**	**50S ribosomal protein L3**
4.347487802	20.3574902	2.30E-67	DN756_RS12480	APC family permease
4.302271957	19.729356	1.97E-17	malK	maltose/maltodextrin ABC transporter ATP-binding protein MalK
4.262147054	19.1881944	1.52E-101	DN756_RS06760	dihydrodipicolinate synthase family protein
4.224799241	18.6978339	1.56E-124	DN756_RS06765	N-acetylmannosamine-6-phosphate 2-epimerase
**4.19204945**	**18.2781664**	**2.51E-110**	**rpmC**	**50S ribosomal protein L29**
**4.191842469**	**18.2755443**	**1.09E-53**	**rpsR**	**30S ribosomal protein S18**
4.124259445	17.4391697	1.37E-91	DN756_RS07545	PTS sugar transporter subunit IIB
**4.058994111**	**16.6678268**	**7.52E-99**	**rpsQ**	**30S ribosomal protein S17**
**4.022704701**	**16.253795**	**4.77E-99**	**rplI**	**50S ribosomal protein L9**
4.01661034	16.1852791	3.03E-87	ompX	outer membrane protein OmpX
**4.006945136**	**16.0772097**	**8.21E-69**	**priB**	**primosomal replication protein N**
**3.999533604**	**15.9948283**	**7.87E-87**	**rpsC**	**30S ribosomal protein S3**
**3.939201347**	**15.3397317**	**1.44E-112**	**rplP**	**50S ribosomal protein L16**
**3.891815541**	**14.8440776**	**2.91E-20**	**rpsB**	**30S ribosomal protein S2**
3.814396217	14.0684962	3.96E-22	speA	biosynthetic arginine decarboxylase
**3.783491797**	**13.7703354**	**2.87E-77**	**rplJ**	**50S ribosomal protein L10**
3.781213946	13.7486108	1.10E-107	DN756_RS06775	N-acetylmannosamine kinase
3.738173255	13.3444992	1.75E-37	htpG	molecular chaperone HtpG
3.731351524	13.2815491	3.40E-28	yidC	membrane protein insertase YidC
3.721580822	13.1919033	2.49E-17	DN756_RS06965	tRNA-Val
**3.691318931**	**12.9180726**	**5.08E-30**	**rpsJ**	**30S ribosomal protein S10**
3.647140184	12.5284861	1.80E-24	DN756_RS19545	SDR family oxidoreductase
3.637044276	12.4411184	7.02E-17	accC	acetyl-CoA carboxylase biotin carboxylase subunit carboxylase C-terminal domain
3.625644565	12.3432	1.14E-67	dnaJ	molecular chaperone DnaJ
**3.619695655**	**12.292408**	**1.03E-57**	**rpsF**	**30S ribosomal protein S6**
3.605060389	12.1683394	4.38E-32	hslV	ATP-dependent protease subunit HslV
**3.584315091**	**11.9946162**	**3.46E-184**	**rplL**	**50S ribosomal protein L7/L12**
3.577533236	11.9383639	3.53E-123	tig	trigger factor
3.57208558	11.8933694	3.48E-19	lpdA	dihydrolipoyl dehydrogenase
**3.563841838**	**11.8256029**	**5.08E-66**	**rplS**	**50S ribosomal protein L19**
3.503267432	11.339361	3.51E-61	DN756_RS12195	TonB-dependent receptor
3.494065924	11.2672687	3.55E-14	DN756_RS06970	tRNA-Val
3.484338798	11.1915565	1.96E-29	malP	maltodextrin phosphorylase
3.461945036	11.0191806	9.74E-30	DN756_RS19555	TonB-dependent hemoglobin/transferrin/lactoferrin family receptor
**3.436664222**	**10.8277698**	**6.09E-93**	**rplA**	**50S ribosomal protein L1**
**3.383401419**	**10.435309**	**7.11E-138**	**rplK**	**50S ribosomal protein L11**
3.366783823	10.3158001	2.82E-35	yiuA	iron-siderophore ABC transporter substrate-binding protein YiuA
3.357159614	10.2472125	9.38E-24	cysK	cysteine synthase A
3.355441448	10.235016	1.17E-23	pyrH	UMP kinase
3.35303223	10.2179383	2.04E-09	cobA	uroporphyrinogen-III C-methyltransferase
3.325103341	10.0220334	1.13E-25	DN756_RS14130	2Fe-2S ferredoxin-like protein

### Ribosomal reporter expression is heightened in exponential phase cells

**Fig 2:**
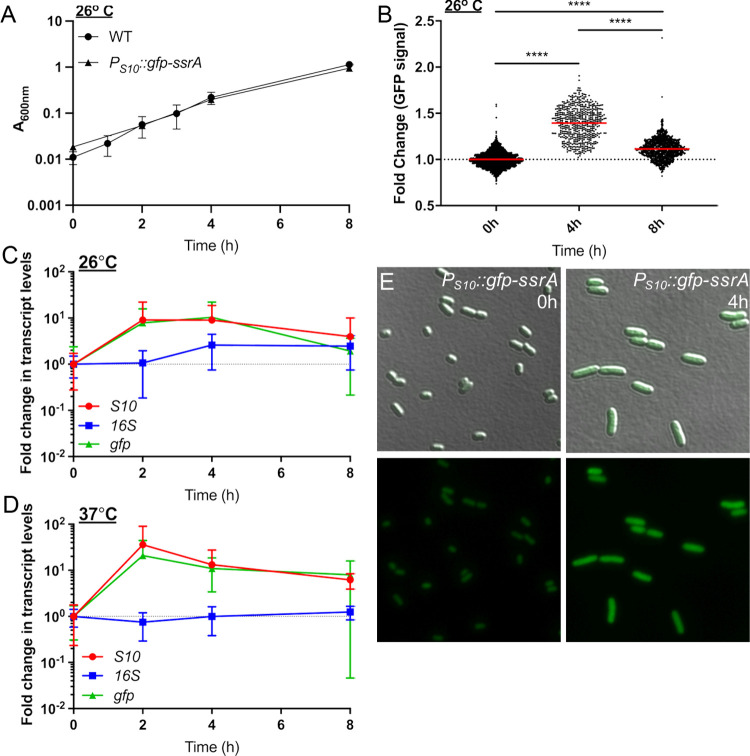
Ribosomal reporter expression is heightened in exponential phase cells. (A) Growth curve of WT and *P*_*S10*_*::gfp-ssrA Y. pseudotuberculosis* strains at 26^o^ C. Absorbance (A_600nm_) detected by plate reader at the indicated timepoints (hours, h). Four biological replicates shown with mean and standard deviation. (B) Single cell fluorescence microscopy used to quantify the fold change in fluorescence of the *P*_*S10*_*::gfp-ssrA* strain at the indicated timepoints, sampled from cultures in (A). Fold change in signal is relative to the mean GFP fluorescence value at 0h, which is shown as a dotted line at a value of 1. Horizontal lines: median values. Data represents 3 biological replicates for each condition. **(C-D)** qRT-PCR to detect *S10, gfp,* and *16S* transcript levels during culture of the *P*_*S10*_*::gfp-ssrA* strain at (C) 26^o^ C or (D) 37^o^ C. RNA was isolated at the indicated timepoints. Data represents 6 biological replicates. (E) Representative images used to quantify single-cell fluorescence corresponding with 0h and 4h timepoints in panel B. Statistics: (B) Kruskal-Wallis one-way ANOVA with Dunn’s post-test, ****p < .0001.

To visualize S10 (*rpsJ*) expression dynamics throughout the cell cycle, an ectopic reporter construct was made using the S10 leader and promoter sequences to drive expression of a ssrA-tagged, destabilized GFP [21,41,42]. The half-life of this destabilized version of GFP is approximately 59 minutes which will allow for visualization of dynamic changes in reporter expression [21]. It is important to note this reporter was generated to detect rapidly growing exponential phase cells based on S10 ribosomal protein expression, in contrast with other approaches that detect ribosomal RNA, which may better represent ribosome abundance [43,44]. After confirming ectopic GFP reporter expression did not affect growth rate (Fig 2A), *P*_*S10*_*::gfp-ssrA* cell populations were sampled at defined timepoints to represent different growth stages, and GFP fluorescent intensity within individual bacterial cells was quantified by fluorescence microscopy (Fig 2B). GFP expression significantly increased as cells transitioned from stationary phase (0h) to exponential phase (4h), and reporter signal significantly decreased at 8h (Fig 2B) as cells began to transition back into stationary phase. These results suggest that the S10 reporter can dynamically detect rapidly growing cells.

To confirm changes in GFP fluorescence reflect changes in S10 ribosomal protein transcript levels, qRT-PCR was performed to quantify transcript levels of *S10* and *gfp,* compared to ribosomal RNA (16S). Transcripts of the housekeeping gene, *rpoC*, were used here for normalization to detect any 16S fluctuation. *P*_*S10*_*::gfp-ssrA* cells were cultured overnight, sampled in stationary phase (0h), sub-cultured, and samples were collected at 2, 4, and 8 hours for RNA isolation. As mentioned above, 26^o^ C is typically used for *Y. pseudotuberculosis* culture, since incubation of *Yersinia* at mammalian body temperatures (37^o^ C) triggers expression of mammalian cell-targeting virulence factors, and prolonged culture can result in loss or inactivation of virulence genes [15,36]. To determine whether the S10 reporter would also detect faster-growing exponential phase cells during culture at 37° C, cells were grown in parallel at 26° C and 37° C. At 26° C, *gfp* and *S10* transcript levels increased between 0 and 2 hours, remained steady at 4h, and began to decline at 8h, while 16S transcripts remained relatively constant (Fig 2D). At 37° C, while 16S transcript levels stayed constant, both *gfp* and *S10* transcript levels peaked at 2h then declined between 4h and 8h, consistent with when bacteria were growing more rapidly (Fig 2E). *gfp* and *S10* transcript levels correlated well at each temperature and throughout the sampled timepoints, suggesting the *P*_*S10*_*::gfp-ssrA* reporter is accurately depicting *S10* transcript levels. Overall, these results indicate the S10 reporter can be utilized to detect rapidly growing exponential phase cells at either temperature.

### Multiple bacterial subpopulations are present within splenic microcolonies

We then sought to utilize the S10 reporter to determine if expression of critical virulence factors, specifically the type-III secretion system (T3SS) alters ribosomal protein expression in individual cells based on S10 reporter expression. In the mouse model of systemic infection, we previously showed that *Y. pseudotuberculosis* at the periphery of microcolonies are in direct contact with neutrophils and express high levels of the T3SS [20]. Bacteria at the center of microcolonies lack host cell contact, and have an intermediate level of T3SS expression, induced by growth at mammalian body temperatures (37^o^ C) [22,34]. We hypothesized that the high levels of T3SS expression within peripheral cells would impact their fitness [14], and could result in decreased S10 reporter expression.

**Fig 3:**
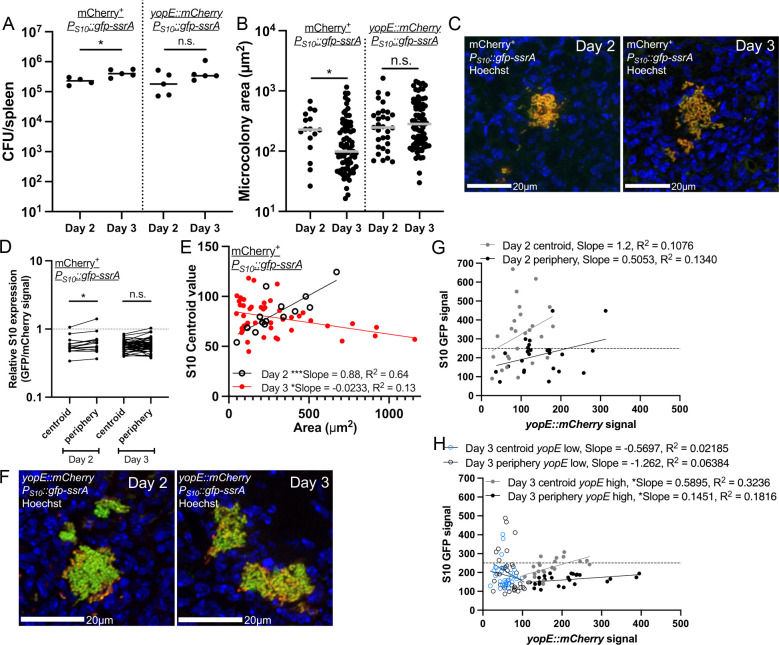
Multiple bacterial subpopulations are present within splenic microcolonies. C57BL/6 mice were inoculated intravenously with the mCherry^+^ *P*_*S10*_*::gfp-ssrA* (constitutive mCherry), *yopE::mCherry P*_*S10*_*::gfp-ssrA*, or *yopE::mCherry gfp-ssrA* (constitutive GFP) strains. (A) CFU/spleen was quantified at days 2 and 3 post-inoculation. Dots: individual mice. (B) Microcolony areas from spleens were quantified by fluorescence microscopy at the indicated timepoints. Dots: individual microcolonies, representing all bacteria visualized within tissues (including single cells). (C) Representative images of microcolonies: day 2 and day 3 mCherry^+^ *P*_*S10*_*::gfp-ssrA*. (D) Reporter expression (ratio GFP/mCherry signal intensity) was quantified at the indicated timepoints and spatial locations (centroid: average of 4 measurements clustered at the geometric centroid of each microcolony, periphery: average of 4 measurements taken every 90^o^ around the microcolony periphery). Horizontal dotted line represents a value of 1, equal amounts of GFP and mCherry signal. (E) Linear regression data indicating slope and R2 value of best fit lines for S10 centroid values compared to microcolony areas at the indicated timepoints. (F) Representative images of microcolonies: day 2 and day 3 *yopE::mCherry P*_*S10*_*::gfp-ssrA*. (G, H) Linear regression data indicating slope and R2 value of best fit lines for GFP compared to mCherry signal of microcolonies at the indicated spatial locations at **(G)** day 2 and **(H)** day 3. Horizontal dotted line at y = 250 separates low and high GFP signal. (H) Microcolonies with low levels of *yopE*::mCherry signal (<100) were analyzed separately (open circles). Dots represent individual mice **(A)** or individual microcolonies (all other panels). Statistics: (A-B) Mann-Whitney; (D) Wilcoxon matched-pairs; ***p < 0.001, *p < .05, ns: not-significant. **(E, G,** H) Significantly non-zero slope indicates correlation between values.

To test this hypothesis, the S10 reporter construct was moved into a strain containing a fluorescent *yopE::mCherry* reporter to detect T3SS expression [20,24]. This is a transcriptional reporter with an internal ribosomal binding site and stable mCherry fluorescent protein gene inserted downstream of *yopE* in the *Yersinia* virulence plasmid. To assess relative S10 reporter expression, we also moved the reporter construct into a strain constitutively expressing mCherry (mCherry^+^) [45]. C57BL/6 mice were infected intravenously, and spleens were harvested at day 2 and day 3 post-inoculation to quantify bacterial colony-forming units (CFUs) and visualize reporter expression by fluorescence microscopy. Based on previous studies, we expected significant bacterial growth between these timepoints, allowing us to differentiate between slower-growing and faster-growing individual cells [21,35]. While the CFUs with the *yopE::mCherry P*_*S10*_*::gfp-ssrA* strain did not significantly increase, CFUs of the mCherry^+^ *P*_*S10*_*::gfp-ssrA* did increase between timepoints (Fig 3A). More microcolonies were detected in cryosections at day 3 (Fig 3B). Microcolonies were significantly smaller in the mCherry^+^ *P*_*S10*_*::gfp-ssrA*-infected tissues at day 3, suggesting we captured a timepoint where larger microcolonies were dissociating to form smaller microcolonies (Fig 3B). Similar day 2 and day 3 microcolony areas in the *yopE::mCherry P*_*S10*_*::gfp-ssrA* infection may suggest smaller microcolonies were formed and had grown by day 3. We expected both infections to progress similarly, but these data may indicate the mCherry^+^ infection is progressing more slowly.

To analyze S10 reporter expression, we first used the mCherry^+^ *P*_*S10*_*::gfp-ssrA* strain to directly compare S10 expression to constitutive mCherry and calculated relative S10 expression based on GFP/mCherry values. The constitutive mCherry signal was used to account for higher bacterial cell density at the geometric centroid of microcolonies vs. the periphery, and baseline cell-to-cell variability in transcriptional and translational activity. Peripheral cells had increased relative S10 expression compared to the centroid at day 2, however there was no spatial difference in S10 expression at day 3 (Fig 3C, 3D). This patterning indicates several things: S10 expression differs from constitutive expression, S10 expression becomes more spatially uniform at day 3, and these data hint that peripheral cells can express higher levels of S10. Interestingly, we also observed a strong positive correlation between centroid S10 levels at the microcolony centroid and areas at day 2, while at day 3, there was an inverse correlation between S10 signal and microcolony areas (Fig 3E). This is consistent with our previous results that larger microcolonies begin to run out of nutrients by day 3 [21,35], and suggests that smaller, newly founded microcolonies exhibit increased S10 expression at day 3 (Fig 3B, 3E).

To explore the relationship between *yopE* and S10 expression, we generated correlation plots of the mCherry and GFP fluorescent signals at the centroid and periphery of individual microcolonies. At day 2, the data suggest that microcolonies express both S10 and *yopE*, which contradicts our hypothesis that high T3SS expression will correlate with low S10 expression (Fig 3F, 3G). However, given the smaller sample size of microcolonies at this timepoint, there was not a correlation between signals. At day 3, the larger number of microcolonies allowed us to also analyze values separately based on levels of *yopE* expression (greater or less than mean fluorescent intensity of 100). *yopE* high cells (MFI ≥ 100) at the centroid and periphery had a weak positive correlation between *yopE* and S10 levels (Fig 3H). In contrast, *yopE* low cells (MFI < 100) displayed some higher levels of S10 expression (MFI > 250), as evidenced by a best-fit line with a negative slope (Fig 3H). These results indicate that multiple populations emerge within the mouse spleen: a surprising population with higher *yopE* expression that retain S10 expression, bacteria with lower *yopE* expression that retain higher S10 expression, consistent with our initial hypothesis, and bacteria with low expression of both. We also noted the high S10 values in this infection set compared to mCherry^+^ (Fig 3E, 3H), which again suggest active growth of microcolonies (Fig 3B).

### T3SS induction slows growth, but cells can support high levels of S10 expression

**Fig 4:**
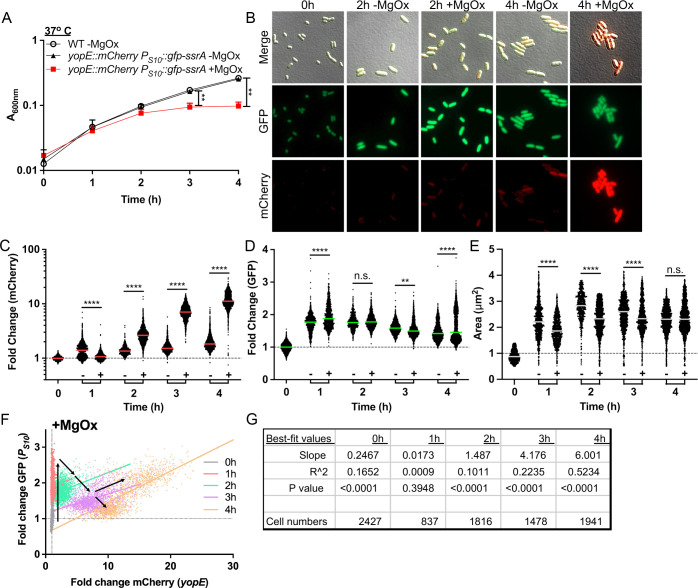
T3SS induction slows growth, but cells can support high levels of S10 expression. WT and *yopE::mCherry P*_*S10*_*::gfp-ssrA* strains were cultured at 37^o^ C in the presence (+, T3SS-induced) or absence (-) of MgOx for the indicated timepoints (hours, h). (A) Growth curve of strains with and without MgOx. Absorbance (A_600nm_) detected by plate reader at the indicated timepoints. Statistics compare the *yopE::mCherry P*_*S10*_*::gfp-ssrA* strain in the presence (+) or absence (-) of MgOx. Mean and standard deviation are shown. (B) Representative fluorescence microscopy images of bacteria from **(A)** immobilized on 1% agarose pads. Fold change in **(C)** mCherry (*yopE::mCherry*) reporter signal and (D) GFP (*P*_*S10*_*::gfp-ssrA*) reporter signal in the absence (-) or presence (+) of MgOx. Values quantified in individual bacteria by fluorescence microscopy. Thresholding was used to select individual cell events, and single cell fluorescence was normalized to the average fluorescent value at 0h (value of 1, represented by dotted line). Each dot: individual cell, horizontal lines: median values. (E) Quantification of single cell bacterial areas (µm2) from samples in panels **(C)** and (D). Horizontal lines: median values. (F) Correlation plot of fold change in single cell mCherry and GFP fluorescence from bacteria in panels **(C-E**) cultured in the presence (+) of MgOx. Black arrows denote trends in fluorescence across timepoints. (G) Linear regression data indicating slope, R2, and significance of the lines of best fit shown in (F). All data represent 3 biological replicates for each strain and condition in this figure. Statistics: (A) Two-way ANOVA with Tukey’s multiple comparison test; (C-E) Kruskal Wallis one-way ANOVA with Dunn’s post-test; ****p < 0.0001, **p < .01, ns: not-significant. (F,G) Significantly non-zero slope indicates correlation between values.

Outside of a mammalian host, T3SS expression can be induced in *Yersinia* cultures by magnesium oxalate (MgOx) treatment resulting in slowed growth [15,38]. This induction strategy involves adding equimolar amounts of MgCl_2_ and sodium oxalate, which dissociate to form NaCl and MgOx [46]. This strategy will add Na^+^ and Mg^2+^ cations in the absence of added calcium (Ca^2+^), and calcium oxalate precipitation of residual calcium will result in low calcium conditions trigger high levels of T3SS expression [15,47]. It is important to note incubation at 37^o^ C induces a low level of T3SS expression, and MgOx addition heightens expression to a growth-inhibited, full induction state. To explore if growth inhibition results in decreased S10 expression, the *yopE::mCherry P*_*S10*_*::gfp-ssrA* reporter strain was grown at 37^o^ C in the presence or absence of MgOx to induce T3SS expression. As expected, growth curves demonstrated significant growth inhibition at 3h and 4h post-MgOx treatment (Fig 4A). T3SS expression was significantly higher in +MgOx treated samples compared to –MgOx between 2h-4h of treatment, correlating well with growth inhibition (Fig 4B, 4C). However, these data suggest T3SS expression may precede growth inhibition, based on the timing of reporter signal detection (2h) and growth inhibition (3h) (Fig 4A, 4C).

Interestingly, while S10 expression generally correlated with growth, the link between S10 expression T3SS expression varied (Fig 4A, 4C, 4D). At the 1h timepoint, untreated cells exhibited higher median T3SS expression, due to temperature, and lower median S10 expression. Over time, MgOx treated cells increased expression of the T3SS and decreased in S10 signal, specifically at 3h post-treatment when growth was also arrested (Fig 4A, 4C, 4D). In contrast, at 4h we observed the median S10 reporter signal was higher in treated cells, largely due to a subset of bacterial cells that retained high S10 expression (Fig 4D). Additionally, MgOx treatment resulted in a smaller median cell area, suggesting a decreased division rate and decreased metabolic activity (Fig 4E) [48]. The difference in median cell size was lost at the 4h timepoint, suggesting untreated and treated cells may be approaching stationary phase.

At the 4h timepoint, we noticed subsets of cells that retained high S10 expression and cells with lower T3SS expression (Fig 4C, 4D). To determine if these were one subpopulation or represented distinct sets of cells, we plotted the fold change of each fluorescent marker for MgOx treated cells. In the first hour of culture S10 expression is strongly induced in all cells, likely capturing their transition out of stationary phase, as seen by a shift up the y-axis (in red) relative to the 0h timepoint (in gray) (Fig 4F). As also seen with transcript levels in Fig 2D, S10 expression levels appear highest at 1h-2h of culture (red, green), and gradually decrease (Fig 4F). After the initial increase, S10 levels generally decreased as T3SS expression increased; evidenced by a shift in the population towards the bottom right of the graph (Fig 4F).

To our surprise, S10 levels did not decrease universally. At 4h post-treatment (in orange) two populations of cells emerged; a population with low S10 and lower T3SS expression and a population with high S10 and high T3SS expression (Fig 4F). This contrasts with our initial hypothesis, and indicates a subpopulation of cells is capable of expressing high levels of both T3SS and ribosomal protein genes (S10). The linear regression analysis was consistent with a positive correlation between the two reporter signals at 4h, based on a significantly non-zero positive slope and R^2^ value of 0.5234 (Fig 4G). There was very little correlation between S10 and T3SS levels in the absence of MgOx based on the slope and R^2^ values of the best-fit lines, likely because T3SS expression was low under these conditions (S2 Fig).

Our experiments in culture indicate that a subset of bacteria exhibit high T3SS activity and high levels of S10 expression, contrary to our initial hypothesis. Subpopulations of bacteria with high T3SS expression and high translational activity have also been observed in cultured *Pseudomonas aeruginosa* [49].

### MgOx treatment is not sufficient to alter S10 reporter activity

We next asked whether MgOx treatment alone, independent of temperature, is sufficient to impact bacterial growth and S10 reporter expression. Bacteria were incubated in the presence or absence of MgOx at 26^o^ C, absorbance measurements were taken, and cells were collected for fluorescence microscopy. The *yopE::mCherry* strain (lacking *P*_*S10*_*::gfp-ssrA*) and non-fluorescent WT strains were used as controls to ensure growth was not impacted by expression of either reporter (*yopE::mCherry* or *P*_*S10*_*::gfp-ssrA*). There were no significant differences, indicating the reporters did not impact growth and that MgOx treatment, without a temperature shift to 37^o^ C, was not sufficient to inhibit bacterial growth (S3A Fig). Fluorescence microscopy indicated MgOx treatment resulted in low levels of *yopE::mCherry* expression (S3 B, C Fig). Levels of T3SS induction were very low relative to MgOx-treated cells at 37^o^ C, and treated cells at 26^o^ C no longer had T3SS induction at 4h post-treatment (S3C Fig). In the absence of significant growth inhibition, S10 levels were minimally impacted by treatment, however MgOx-treated cells had lower S10 expression at 3h post-treatment (S3 D Fig), and we observed a decrease in cell size 2h-4h after MgOx treatment (S3 E Fig). The decreased cell size could suggest that MgOx treatment has other impacts on cell physiology (S3E Fig).

### Lack of T3SS expression was associated with lower S10 expression

To further assess the possible link between T3SS expression and S10 reporter expression, we transformed the *P*_*S10*_*::gfp-ssrA* reporter plasmid into a plasmid-cured *Y. pseudotuberculosis* strain lacking the virulence plasmid (P(-)) [36,47], a 68kb extrachromosomal plasmid that encodes for the T3SS structural proteins and associated effector proteins [36,50]. It is important to note there are additional genes encoded on the virulence plasmid, notably *yadA*, a temperature-regulated adhesin and known virulence factor that promotes injection of T3SS effector proteins into host cells [31]. We first utilized the P(-) strain for culture-based experiments to assess the role of T3SS expression, since we expected the impact of YadA to be minimal under these conditions.

**Fig 5:**
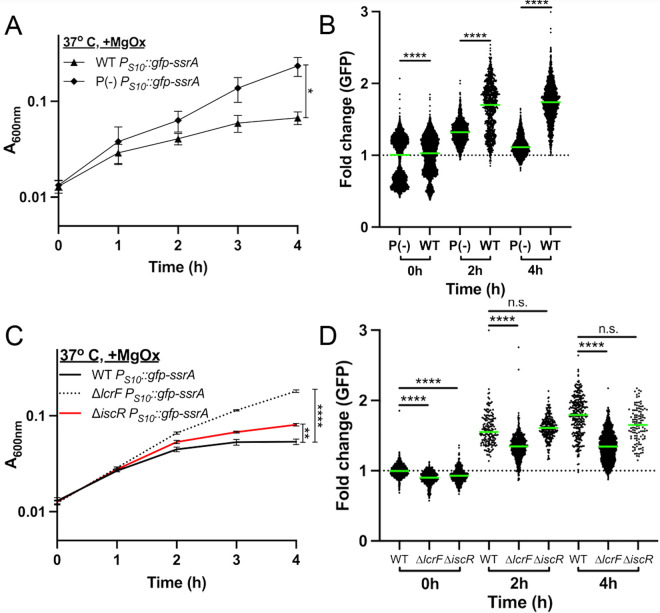
Lack of T3SS expression was associated with lower S10 expression. WT *P*_*S10*_*::gfp-ssrA,* and P(-) *P*_*S10*_*::gfp-ssrA* (virulence plasmid-cured) strains were cultured at 37^o^ C in the presence (+) of MgOx for the indicated timepoints (hours, h). (A) Growth curve of strains, absorbance (A_600nm_) was detected by plate reader at the indicated timepoints. Mean and standard deviation are shown. (B) Fluorescence microscopy to quantify single cell fold change in GFP (*P*_*S10*_*::gfp-ssrA*) reporter signal. Single cell fluorescence was normalized to the average fluorescent value of the WT strain at 0h (value of 1, represented by dotted line). Each dot: individual cell, horizontal lines: median values. (C) WT *P*_*S10*_*::gfp-ssrA*, ∆lcrF *P*_*S10*_*::gfp-ssrA*, and ∆*iscR P*_*S10*_*::gfp-ssrA* were cultured at 37^o^ C in the presence (+) of MgOx. Growth curve of strains, absorbance (A_600nm_) was detected by plate reader at the indicated timepoints. Statistics compare the WT strain to other strains. Mean and standard deviation are shown. (D) Fluorescence microscopy to quantify single cell fold change in GFP reporter signal. Single cell fluorescence was normalized to the average fluorescent value of the WT strain at 0h (value of 1, represented by dotted line). Each dot: individual cell, horizontal lines: median values. All data represent 3 biological replicates for each strain and timepoint in this figure. Statistics: (A, C) Two-way ANOVA with Tukey’s multiple comparison test; (B, D) Kruskal Wallis one-way ANOVA with Dunn’s post-test; ****p < 0.0001, **p < .01, *p < .05, ns: not-significant.

WT *P*_*S10*_*::gfp-ssrA* and P(-) *P*_*S10*_*::gfp-ssrA* strains were treated with MgOx and grown at 37^o^ C. As expected, the P(-) *P*_*S10*_*::gfp-ssrA* strain had significantly higher absorbance values than WT *P*_*S10*_*::gfp-ssrA* after 4h culture, indicating the presence of the virulence plasmid impaired growth (Fig 5A). To determine if differences in growth correlated with changes in S10 levels, we detected S10 reporter expression by fluorescence microscopy. The WT *P*_*S10*_*::gfp-ssrA* and P(-) *P*_*S10*_*::gfp-ssrA* strains had similar levels of S10 expression at 0h, although the WT strain was slightly higher (Fig 5B). Surprisingly, S10 expression levels continued to increase in the WT strain, and WT exhibited significantly higher S10 levels than the P(-) strain at both 2h and 4h, despite significantly slower growth (Fig 5A, 5B). To better assess the impact of T3SS expression on S10 expression, we completed experiments with ∆*lcrF* and ∆*iscR* strains, which, respectively, lack and exhibit lower T3SS expression under these conditions [51,52]. We observed the expected growth patterns with these strains at 37^o^ C + MgOx, where the WT strain exhibited significantly slower growth than both mutants, and the ∆*lcrF* strain, which lacks T3SS expression, exhibited the most rapid growth (Fig 5C). Surprisingly once again, we detected significantly higher S10 levels in the WT strain compared to the ∆*lcrF* strain lacking T3SS expression, and no difference at the 2h and 4h timepoints between the WT and ∆*iscR* strains (Fig 5D). To determine if this apparent discrepancy in the growth curve and S10 levels was due to differences in cell morphology, specifically if smaller cells were exhibiting higher S10 signal because the GFP molecules are condensed in a smaller space, we quantified the single cell areas of each strain. While WT cells were significantly smaller than the mutants at 2h, there was no difference at 4h, a timepoint that also showed different S10 values (S4 Fig). These data strongly indicate that T3SS expression can result in both growth arrest and retention of high S10 expression levels, and provides some explanation as to why we detected cells expressing both T3SS and S10 in the mouse model and in culture.

### Droplet-based microfluidics can be used to model clustered bacterial growth

One limitation of *in vivo* infection models is the inability to track growth and reporter expression profiles over time. In contrast, culture-based experiments can enable kinetic analyses, but lack the clustered spatial architecture of bacteria seen within the host environment. To circumvent these issues, droplet-based microfluidics can be used to generate local agar environments that force bacteria to grow in tightly-clustered colonies, as they would within host tissues [20,53]. Here, we employed microfluidic droplets to observe growth and reporter expression over time during incubation at 26^o^ C and 37^o^ C, and at 37^o^ C in the presence and absence of MgOx. Agarose-based microfluidics droplets were generated as previously described, where liquid and oil phases intersect to generate oil-encapsulated spherical droplets, and an appropriate bacterial density in the liquid phase was used to ensure a majority of droplets were seeded with a single bacterial cell [53]. Oil was removed from the exterior of droplets to allow free diffusion of molecules into and out of the droplets during culture [53]. We hypothesized that MgOx addition during culture at 37^o^ C would result in high levels of T3SS induction, growth inhibition, and may result in either higher or lower S10 levels. Based on results in culture, we hypothesized that incubation at 37^o^ C would not significantly delay droplet growth relative to 26^o^ C, since this should result in intermediate T3SS induction.

**Fig 6:**
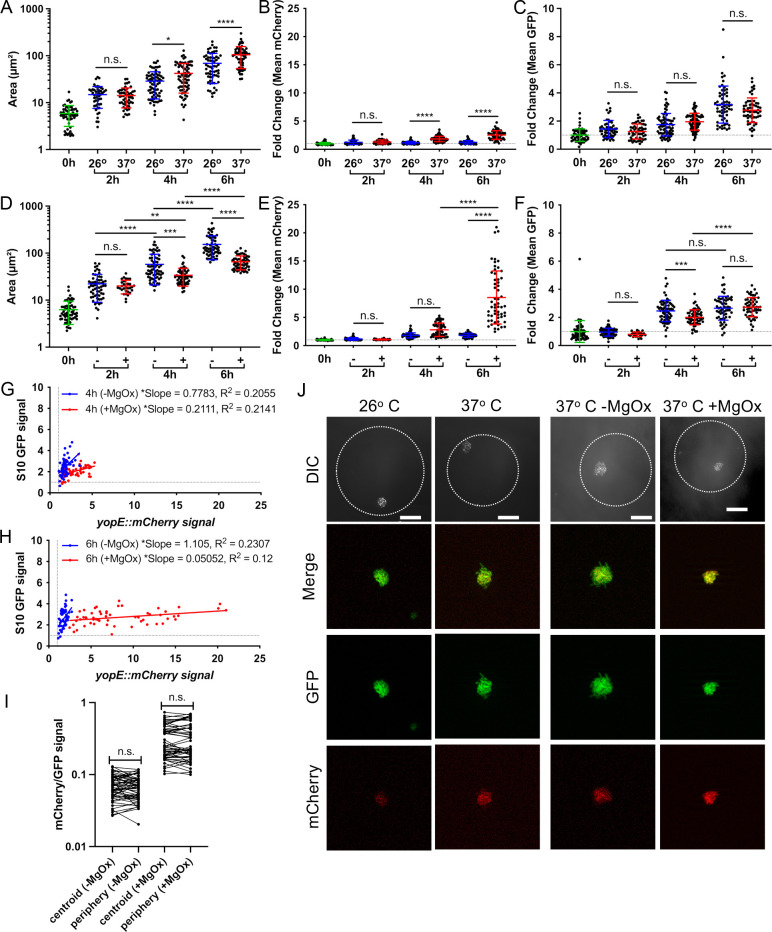
Droplet-based microfluidics can be used to model clustered bacterial growth. The *yopE::mCherry P*_*S10*_*::gfp-ssrA* strain was encapsulated into droplets and cultured at the indicated temperatures in the presence (+) or absence (-) of MgOx. At the indicated times aliquots were taken and bacterial growth and reporter expression were quantified by fluorescence microscopy. Growth at 26^o^ C was compared to 37^o^ C, and **(A)** area (µm2), **(B)** fold change in mCherry signal (relative to 0h), and **(C)** fold change in GFP signal (relative to 0h) were quantified. Horizontal dotted lines represent a value of 1, the initial average value at time 0h. Growth at 37^o^ C in the presence (+) or absence (-) of MgOx was compared, and **(D)** area (µm2), **(E)** fold change in mCherry signal (relative to 0h), and **(F)** fold change in GFP signal (relative to 0h) were quantified. **(G, H)** Linear regression data indicating slope and R2 value of best fit lines for GFP compared to mCherry signal of microcolonies grown **(G)** 4h or **(H)** 6h at 37^o^ C, (+) or (-) MgOx. **(I)** mCherry/GFP signal ratios are shown at the indicated spatial locations for microcolonies grown -/ + MgOx. **(J)** Representative images of microcolonies grown within droplets under the indicated conditions, all images represent 6h timepoints. Differential Interference Contrast (DIC) shown alongside GFP, mCherry, and merged fluorescent channels. Dotted line outlines the periphery of each droplet. Scale bar: 20µm. All data represent 2 biological replicates for each condition in this figure. Mean and standard deviation are shown. Each dot represents an individual microcolony. Statistics: **(A-F)** Two-way ANOVA with Tukey’s multiple comparison test; **(G, H)** Linear regression, significantly non-zero slope indicates correlation between values; **(I)** Wilcoxon matched-pairs; ****p < 0.0001, ***p < .001, **p < .01, *p < .05, ns: not-significant.

We first explored if temperature impacted growth by encapsulating the *yopE::mCherry P*_*S10*_*::gfp-ssrA* strain into droplets and culturing at 26^o^ C or 37^o^ C. Cultures were sampled every two hours to quantify bacterial growth, T3SS reporter signal, and S10 reporter signal by fluorescence microscopy. Bacterial clusters grew at both temperatures, but were significantly larger at 37^o^ C (Fig 6A). While microcolonies had significant T3SS induction at 4h and 6h of growth at 37^o^ C (Fig 6B), there was no change in S10 reporter signal between conditions (Fig 6C). These results corroborate planktonic cell culture experiments where growth at 37^o^ C moderately induces T3SS expression but does not reduce growth rate (Fig 4A-C).

While temperature would be sensed by the entire population during infection, local chemical mediators, such as those released from surrounding neutrophils or monocytes [20], can create chemical gradients experienced most strongly by peripheral cells in the microcolony. In the droplet system, we would expect MgOx to result in uniform low calcium conditions. During MgOx treatment at 37^o^ C, we observed significant droplet growth inhibition at 4h and 6h post-treatment (Fig 6D) and high levels of T3SS induction (Fig 6E) after 6h of culture. These results mirror experiments in Fig 4 in bacterial media, where strong T3SS expression results in growth inhibition (Fig 4A, 4C). As also seen in previous figures, S10 expression did not directly correlate with growth inhibition. At the 4hr timepoint S10 expression was significantly lower in the growth inhibited +MgOx group, but S10 levels were similar at the 6h timepoint (Fig 6F). Interestingly, correlation plots clearly showed that T3SS expression increased between 4h and 6h without an impact on S10 levels, again uncoupling T3SS expression, growth arrest, and S10 levels (Fig 6G,6H).

To determine if the local position in the droplet community impacts reporter expression, we quantified the centroid and peripheral fluorescence values within individual droplets at the 6h timepoint, when T3SS reporter signal was highest. While we have consistently seen a significant increase in T3SS signal at the periphery of microcolonies within host tissues (Fig 3F) [20], we did not observe spatial differences in reporter signal within droplets (Fig 6I, 6J). This ready diffusion of compounds could be beneficial for future applications of this technology, where researchers could generate uniform microenvironments within a single droplet or even droplet configurations. These results also suggest that contact with the agarose matrix of droplets, which could mimic surface contact, is not sufficient to mimic neutrophil contact within host tissues.

### T3SS induction reduces gentamicin susceptibility, but S10 expression predicts survival

**Fig 7:**
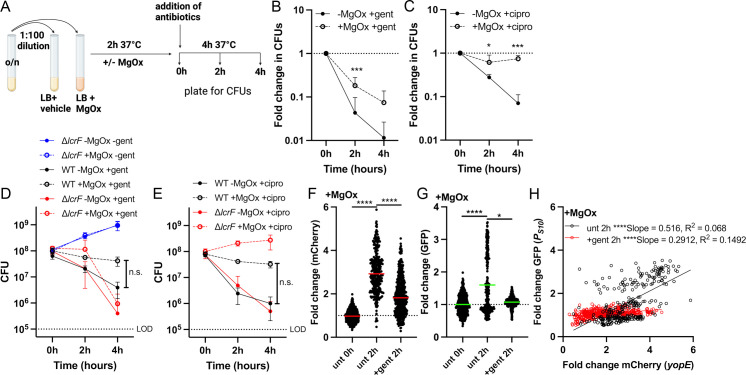
T3SS induction reduces gentamicin susceptibility, but S10 expression predicts survival. Strains were grown in the presence (+) or absence (-) of MgOx for 2h at 37o C. Antibiotics were added (gentamicin: gent, ciprofloxacin: cipro) and strains were cultured an additional 4h. Samples were taken at 0h (addition of antibiotics), 2h, and 4h to quantify CFUs or quantify fluorescence by microscopy. (A) Experimental design. Image Created in BioRender. Cotten, **K.** (2025) https://BioRender.com/b41q462 **(B)** *yopE::mCherry P*_*S10*_*::gfp-ssrA* strain + /- MgOx, gent treatment. **(C)** *yopE::mCherry P*_*S10*_*::gfp-ssrA* strain + /- MgOx, cipro treatment. Fold change in CFUs is calculated by normalizing the number of viable bacteria at 2h and 4h to the number of viable bacteria at 0h. (D) Comparison between non-fluorescent WT and ∆*lcrF* strains, + /- MgOx, gent treatment. Raw CFU values are shown. LOD: limit of detection (horizontal dotted line). (E) Comparison between non-fluorescent WT and ∆*lcrF* strains, + /- MgOx, cipro treatment. Raw CFU values are shown. Fold change in **(F)** mCherry (*yopE*) and (G) GFP (S10) signal from cells represented in **(B)** was calculated by normalizing the single cell fluorescence to the average fluorescent untreated (unt) value at 0h (value of 1, dotted line). Untreated 0h, 2h, and gentamicin 2h are shown. Each dot: individual cell, horizontal lines: median values. Dead cells were excluded using the fixable blue dead cell stain. (H) Linear regression data indicating slope and R2 value of best fit lines for GFP compared to mCherry signal of bacteria in the presence of MgOx, either untreated (unt) 2h or gent treatment 2h. Statistics: (B-E) Two-way ANOVA with Tukey’s multiple comparison test, statistics shown represent comparisons + /- MgOx; (F, G) Mann-Whitney; (H) Linear regression, significantly non-zero slope indicates correlation between values; ****p < 0.0001, ***p < .001, *p < .05.

We hypothesized that the growth arrest observed with MgOx treatment may be sufficient to decrease antibiotic susceptibility, based on known associations between slowed growth, reduced metabolic activity, and decreased antibiotic susceptibility [54,55]. To test this hypothesis, we cultured the *yopE::mCherry P*_*S10*_*::gfp-ssrA* strain in the presence and absence of MgOx, then exposed bacteria to either gentamicin or ciprofloxacin (Fig 7A). These antibiotics were chosen because they are bactericidal, albeit with distinct drug targets, the ribosome and DNA gyrase, respectively. MgOx treatment significantly reduced the susceptibility of *Y. pseudotuberculosis* to gentamicin, as evidenced by increased survival during gentamicin exposure (Fig 7B). MgOx treatment was also sufficient to promote tolerance to ciprofloxacin (Fig 7C). However, it has been well-established that the presence of metal cations, including Mg^2+^, can reduce ciprofloxacin activity [56]. To determine whether altered antibiotic susceptibility was linked to T3SS expression, we performed additional experiments with a ∆*lcrF* strain, which lacks T3SS induction [52]. While differences between the groups didn’t reach statistical significance, the ∆*lcrF* strain had similar growth kinetics in the presence and absence of MgOx and ∆*lcrF* gentamicin sensitivity was similar in the presence or absence of MgOx (Fig 7D), suggesting that the reduced sensitivity of the WT strain to gentamicin was linked to T3SS induction. In contrast, ∆*lcrF* had similar ciprofloxacin sensitivity compared to the WT strain in the absence of MgOx, and in the presence of MgOx, the ∆*lcrF* continued to replicate during ciprofloxacin exposure, consistent with resistance (Fig 7E). This suggests that reduced ciprofloxacin sensitivity was instead due to cation interaction with the antibiotic, and not T3SS expression.

To eliminate the impact of magnesium cations on antibiotic activity, we also performed experiments in M9 minimal media, which are conditions known to induce T3SS expression [51]. While we observed T3SS expression during culture in M9, we did not observe WT growth arrest relative to the ∆*lcrF* strain (S5A Fig), and consistent with this, we also did not observe changes to antibiotic susceptibility (S5B Fig). It is important to note we prepared our M9 media using the protocol in Miller JH 1972 [57] instead of the recipes used in Cheng LW et al. [58] and Miller HK et al. [51], and the calcium in our media explains why we did not observe growth arrest.

To determine the reporter expression profile of bacterial survivors following gentamicin treatment, we performed fluorescence microscopy with the *yopE::mCherry P*_*S10*_*::gfp-ssrA* strain after 2h of gentamicin treatment. 2h was chosen because we observed significant changes in viability (Fig 7B) and because this timepoint correlates with the 4h MgOx timepoint of Fig 4 and Fig 5. We quantified fluorescent reporter expression of surviving, viable bacterial cells by incorporating a live/dead fluorescent dye to exclude non-viable cells from our analyses (see Methods). When compared with untreated samples (2h), bacteria that survived gentamicin treatment had significantly lower mCherry (Fig 7F) and lower S10 (Fig 7G) reporter signal. A comparison of the two reporter signals within individual cells indicated a specific loss of cells expressing high levels of S10, some of which also had high *yopE::mCherry* expression (Fig 7H).

### Gentamicin treatment selects for surviving bacteria with reduced S10 expression in the mouse spleen

**Fig 8:**
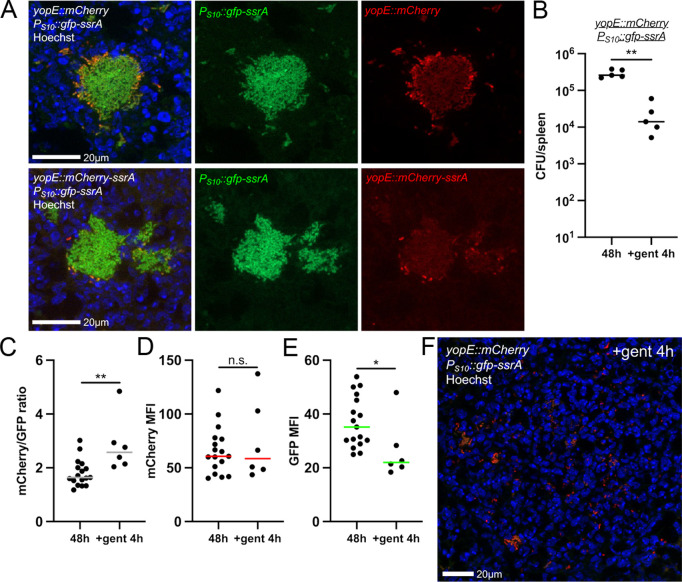
Gentamicin treatment selects for surviving bacteria with reduced S10 expression in the mouse spleen. C57BL/6 mice were inoculated intravenously with the *yopE::mCherry P*_*S10*_*::gfp-ssrA* or *yopE::mCherry-ssrA P*_*S10*_*::gfp-ssrA* strains. At 48h p.i., spleens were harvested from a subset of mice to represent the time of treatment (48h). Additional sets of mice were injected intraperitoneally with 40mg/kg gentamicin, and spleens were harvested 4h post-treatment (+gent 4h) (A) Representative images at 48h p.i. (untreated). (B) CFU/spleen at 48h compared to 4h gent treatment. Dots: individual mice. (C) Reporter expression (ratio mCherry/GFP signal intensity) was quantified at the indicated timepoints. Dots: individual microcolonies. **(D)** mCherry mean fluorescent intensity (MFI) across individual microcolonies. Dots: individual microcolonies. (E) GFP mean fluorescent intensity (MFI) across individual microcolonies. Dots: individual microcolonies. (F) Representative image at 4h post-treatment. Scale bars: 20µm. Statistics: (B-E) Mann-Whitney; **p < .01, *p < .05, ns: not-significant.

To determine if gentamicin selects for surviving bacterial cells with lower S10 levels during infection, we set-up a gentamicin treatment mouse model. Experiments were performed with the *yopE::mCherry P*_*S10*_*::gfp-ssrA* strain, and in parallel, we performed experiments with a *yopE::mCherry-ssrA P*_*S10*_*::gfp-ssrA* strain containing a destabilized version of the mCherry T3SS reporter. The reporter expression patterns were very similar with the two strains, although the overall magnitude of signal was lower with the destabilized reporter, and fewer cells seemed to express very high levels of the T3SS (Fig 8A). Given the similar reporter patterns, we tested gentamicin sensitivity with the stable T3SS reporter (*yopE::mCherry P*_*S10*_*::gfp-ssrA*) strain. We harvested untreated spleens at the 48h timepoint, then treated a subset of mice at 48h with a single intraperitoneal injection of 40mg/kg gentamicin, which should result in rapid systemic delivery of inhibitory concentrations of gentamicin, meeting or exceeding 10µg/ml (MBC_99_) [59]. Spleens were harvested 4h post-treatment and we found gentamicin treatment significantly reduced the bacterial load at this timepoint (Fig 8B). We processed tissues for fluorescence microscopy and specifically analyzed surviving bacteria based on retention of nucleic acid (Hoechst) signal. We observed significantly higher mCherry/GFP ratios in treated tissues (Fig 8C), which was not due to a change in mCherry (*yopE*) MFI (Fig 8D), but instead was specifically due to lower GFP (S10) MFI (Fig 8E). Microcolonies were significantly disrupted by gentamicin treatment based on images before (Fig 8A) and after (Fig 8F) treatment. High levels of mCherry expression were detected within surviving bacteria (Fig 8F), however the image quantification shows *yopE* (mCherry) levels in treated mice (+gent 4h) are similar to untreated (48h), and instead surviving bacteria specifically have low S10 (GFP) expression.

## Discussion

In this study, we sought to understand the impact of T3SS-induced growth arrest on exponential phase markers, specifically S10 ribosomal protein expression, and determine whether reduced expression of ribosomal protein genes correlated with reduced antibiotic susceptibility. We generated a fluorescent transcriptional reporter to detect levels of expression of the S10 ribosomal protein operon and found high, sustained levels of T3SS expression were required for growth arrest and altered expression of S10. Interestingly, the bacterial population diverged after T3SS induction, and we observed both high and low S10 expressing populations in the mouse spleen, in an agarose droplet model of clustered bacterial growth, and during planktonic growth in culture. Importantly, we show high levels of expression of the T3SS are sufficient for growth arrest, expression of the T3SS promotes antibiotic tolerance, and bacterial cells that survive antibiotic treatment have lower levels of S10 expression.

The reporter construct generated here was used to mark cells that had a rapid-growth phenotype compared to slower-growing bacterial cells. While this construct utilized a promoter upstream of the *rpsJ*/S10 gene, it remains unclear if this reporter detects altered ribosome levels. A previous study by Manina et al. used a similar approach, but instead generated a fluorescent transcriptional reporter for ribosomal RNA in *Mycobacterium tuberculosis*, which may better indicate overall ribosome numbers since rRNA levels typically dictate ribosome biogenesis rates [43]. Interestingly, in studies focused on regulation of ribosomal components, it was shown that the *rpsJ*/S10 promoter has unique regulation relative to other ribosomal proteins, and can instead mirror transcript levels of rRNA due to DksA- and ppGpp-dependent transcriptional regulation of *rpsJ* expression [40,60]. The ppGpp-dependent regulatory element found in the leader sequence is included in our reporter, but it is important to note that the first ten amino acids of RpsJ were not included. These amino acids form a hairpin structure that is important for translational regulation [60], thus our reporter is indicating transcriptional activity.

Similar to our results with *Y. pseudotuberculosis*, populations of *M. tuberculosis* exhibit a great deal of heterogeneity in expression of ribosomal genes, which can be amplified under stressful conditions, including infection of the mouse lung and stationary phase culture [43]. Following administration of isoniazid in culture, the majority of surviving bacteria expressed higher levels of rRNA, despite an enrichment in the proportion of nongrowing cells [43]. We also detected heterogeneity within the surviving bacterial population following gentamicin treatment, but overall found increased T3SS and decreased S10 expression in surviving cells, which may better align with results in *Salmonella* showing metabolically-active cells with decreased growth rates have the highest survival rates during antibiotic exposure [61–63]. Interestingly, results in the mouse model of gentamicin treatment indicated that low S10 expression was a better predictor of bacterial survival, which may suggest limited impacts of T3SS expression on metabolic activity at the day 2 (48h) timepoint we chose for treatment.

Previous studies utilizing *Salmonella* have shown that T3SS expression is sufficient to decrease bacterial growth rates, indicating T3SS expression can come at a fitness cost [1,64,65]. Similar results have been shown with *Yersinia* in culture, where bacterial cells injecting T3SS effectors are growth arrested [14]. Here we sought to determine if T3SS-induced growth arrest impacted ribosomal protein expression, since we detected reduced transcript levels of ribosomal protein genes in slower growing, stationary phase *Y. pseudotuberculosis* cultures. Additional studies are needed to compare stationary phase and T3SS-induced growth arrest, but based on our experiments, we predict T3SS induction generates a pronounced, distinct, expression profile with specific effects on cell division machinery and metabolism. It will also be important to separate effects of cations generated during T3SS induction from effects of expression of the T3SS itself, which could be uncoupled using the ∆*lcrF* deletion strain described here [52]. We also attempted to induce T3SS expression with M9, but unfortunately did not detect growth arrest of the WT strain relative to ∆*lcrF* due to low levels of calcium present, and subsequently did not detect antibiotic susceptibility differences under these conditions. Given the data in Fig 5D, the lower S10 expression in ∆*lcrF* would predict these cells would more readily survive gentamicin treatment, and consistent with this, the ∆*lcrF* strain showed survival at the 2h timepoint (Fig 7D). It is unclear what then drives this shift to ∆*lcrF* susceptibility at the 4h timepoint, but this could be explored further to determine if the intracellular gentamicin concentrations in the WT and ∆*lcrF* strains differ.

In *Salmonella* systems, it has also been shown that T3SS expression is linked to decreased antibiotic susceptibility [1,2,66]. In *Yersinia*, much is known about regulation of the T3SS at the molecular level, and it is well appreciated that dynamic expression of this system is required for virulence [22,51,67], and so regulation of the system was not a focus of this study. Instead we sought to link altered T3SS expression levels with differences in S10 reporter signal as a measure of growth rate, and determine whether this impacts changes in antibiotic susceptibility. We also chose to focus on bactericidal antibiotics; the ribosome-targeting gentamicin, and DNA gyrase-targeting ciprofloxacin, to determine the relationship between T3SS expression, S10 expression levels, and antibiotic susceptibility. Our finding that gentamicin treatment resulted in survival of cells with lower levels of S10 reporter expression is consistent with the model that cells with lower metabolic activity were better able to survive antibiotic treatment. Although gentamicin sensitivity can be linked to differences in drug uptake, it has also been recently shown that susceptibility can also depend on ribosomal protein levels, consistent with our results [68].

While some of our results here suggest a relationship between T3SS expression and S10 expression, it is now also apparent that T3SS expression can occur independent of changes in S10 expression levels. It is possible that the length of time of T3SS induction may be a factor, but our data also suggest that cells move through a stage where both signals are high. Ultimately, our results indicate that the S10 reporter can be used to mark rapid growth, and a reduction in S10 signal correlates with reduced antibiotic susceptibility. This information can be harnessed in future studies to better understand changes in antibiotic susceptibility, and the factors that dictate bacterial survival following antibiotic exposure.

## Materials and methods

### Ethics statement

All animal studies were approved by the Institutional Animal Care and Use Committee of Johns Hopkins University, protocol number: MO22H322. Animal studies and procedures were performed in compliance with Animal Welfare Act regulations and Public Health Service (PHS) Policy.

### Bacterial strains & growth conditions

The WT *Y. pseudotuberculosis* strain, IP2666, was used throughout [20,69]. For *in vitro* experiments with fluorescent reporters, bacteria were grown in LB broth overnight (16h) at 26^o^ C with rotation, sub-cultured 1:100, and then incubated at either 26^o^ C or 37^o^ C with rotation in the absence (-MgOx) or presence of magnesium oxalate (+MgOx: 20mM MgCl_2_ and 20mM sodium oxalate [15,33]) to induce T3SS expression for the indicated timepoints. Equivalent volume (200µl) dH_2_O was added to -MgOx tubes. Bacterial growth was assessed based on absorbance at 600nm using a Synergy H1 microplate reader (Biotek). For mouse infection experiments, bacteria were grown overnight (16h) to post-exponential phase in 2xYT broth (LB, with 2x yeast extract and tryptone) at 26^o^ C with rotation as previously described [20]. The *P*_*S10*_*::gfp-ssrA* reporter was expressed from the low copy pMMB67EH plasmid; all cultures with strains containing this construct were supplemented with carbenicillin (100ug/ml). Our M9 media was prepared using the Miller JH 1972 protocol [57]: 6g/L Na_2_HPO_4_, 3g/L KH_2_PO_4_, 0.5g/L NaCl, and 1g/L NH_4_Cl were dissolved in distilled H_2_O and autoclaved, then supplemented with filter sterilized components at final concentrations of: 1mM MgSO_4_, 0.2% glucose, 0.2% casamino acids, and 0.1mM CaCl_2_.

### RNA isolation and qRT-PCR

Bacterial cells were grown in LB broth for the indicated time points, pelleted, resuspended in Buffer RLT (QIAGEN) + ß-mercaptoethanol, and RNA was isolated using the RNeasy kit (QIAGEN). DNA contamination was eliminated using the on-column DNase digestion kit (RNase-free DNase set, QIAGEN). RNA was reverse transcribed using the Protoscript II First Strand cDNA synthesis kit (NEB). qRT-PCR reactions were prepared with 0.5 µM of forward and reverse primers added to cDNA samples and amplification using SYBR Green (Applied Biosystems). *rpoC* was used as an endogenous control since 16S transcript levels were detected as a target gene. Control samples were prepared that lacked reverse transcriptase to confirm DNA was eliminated from samples. Reactions were carried out using the StepOnePlus Real-Time PCR system (Applied Biosystems), and relative comparisons were obtained using the ∆∆C_T_ or 2^-∆Ct^ method. Kits were used according to manufacturers’ protocols.

### RNA-seq

Overnight cultures (16h, stationary phase) were sub-cultured 1:100 into fresh LB and grown an additional 4h at 26^o^ C with rotation to generate exponential phase samples (A_600nm_=0.2). Between 3 x 10^8^-1 x 10^9^ bacterial cells were collected from each culture and RNA was isolated as described above. Purified RNA was shipped to Novogene (Novogene Corporation Inc), for library preparation, sequencing, and bioinformatic analysis. Sequencing reads were aligned to IP2666 genome assembly GCA_003814345.1. The DESeq2 method of pairwise comparisons between treatment groups [70] was used to determine significant differences in transcript levels between stationary and exponential phase cells. An adjusted p-value of 0.05 was considered significant, and hits were determined based on ≥2 fold change, and transcripts with less than 100 mean reads under both conditions were excluded. Full datasets are in S1 and S2 Tables. KEGG pathway analyses were utilized to determine the biological pathways overrepresented in exponential phase compared to stationary phase.

*Generation of reporter strains* The following *Y. pseudotuberculosis* strains were previously described: the strain lacking the virulence plasmid (P(-)) [71], the *yopE::mCherry* strain [20, 24], the mCherry^+^ strain [45],and the ∆*lcr* and ∆*iscR* strains [51,52]. The *P*_*S10*_*::gfp-ssrA* reporter was constructed by fusing the *P*_*S10*_ promoter to *gfp-ssrA* by overlap extension PCR and ligating this fragment into the multiple cloning site of the low copy pMMB67EH plasmid. *P*_*S10*_*::gfp-ssrA* strains were constructed by transformation of this plasmid into *Y. pseudotuberculosis* strains using a previously described protocol [72]. The constitutive GFP construct in pACYC184 was used as a template for mutagenesis to generate the *gfp-ssrA* construct [20], which was constructed by inserting a ssrA-tag at the 3’ end of *gfp* between the last coding amino acid and the stop codon [21].

### Fluorescence microscopy: bacteria

Bacteria were pelleted, resuspended in 4% paraformaldehyde (PFA) and incubated overnight at 4^o^ C for fixation. PFA was removed and bacteria were resuspended in PBS prior to imaging. Agarose pads were prepared to immobilize bacteria for imaging by solidifying a thin layer of 25µl 1% agarose in PBS between a microscope slide and coverslip [73]. Once solidified, coverslips were removed, bacteria were added, coverslips were replaced, and bacteria were imaged with a 63x oil immersion objective, using a Zeiss Axio Observer 7 (Zeiss) inverted fluorescent microscope with XCite 120 LED boost system and an Axiocam 702 mono camera (Zeiss). Volocity image analysis software was used to quantify the fluorescent signal associated with individual bacterial cells, as recently described [73]. Briefly, individual bacterial cells were selected as objects using the green fluorescent channel, previewed to view the objects selected, then thresholding was used to ensure only individual cells were included in the analysis. WT non-fluorescent cells were grown in parallel for all experiments to detect baseline background fluorescence.

### Antibiotic susceptibility experiments

Overnight cultures were diluted 1:100 into fresh LB treated with MgOx (20mM MgCl + 20mM NaOx) or the same volume of H_2_O (-MgOx) and grown at 37°C to induce the T3SS. After 2h antibiotics were added (gentamicin: 10µg/mL (MBC_99_), ciprofloxacin: 0.1µg/mL (MBC_90_)); this represents the 0h timepoint. Samples were taken at 0h, 2h, and 4h to plate for CFUs and for staining with the Live/Dead Fixable Blue Dead Cell Stain Kit (Invitrogen). After washing in PBS, bacterial samples were stained for 30 minutes, protected from light, and washed in PBS before fixation in 4% PFA. Fixed samples were imaged as described above on agarose pads, and cells with detectable blue fluorescence (non-viable) were excluded from single cell analyses. Experiments completed in M9 media followed the same experimental outline of dilution into M9, and antibiotics were either added after 2h or 3h culture, as denoted in the Figure. For these experiments, the *yopE::mCherry* strain was grown in parallel as a control to determine when cells began expressing the T3SS.

### Murine model of systemic infection

Six to eight-week old female C57BL/6 mice were obtained from Jackson Laboratories (Bar Harbor, ME). Mice were injected intravenously into the tail vein with 10^3^ bacteria for all experiments. At the indicated timepoints post-inoculation (p.i.), spleens were removed. ½ of each spleen was homogenized and plated to determine CFU/spleen. Intact tissue (remaining ½ spleen) was processed for fluorescence microscopy as described below.

### Murine model of gentamicin treatment

Inoculations were performed as described above, and at 48 hours p.i., mice were either injected intraperitoneally with 40mg/kg gentamicin or sacrificed to take an untreated timepoint at the point of treatment. This dosage and route was chosen because it should result in a serum concentration between 10–20µg/ml within 20 minutes after injection [59]. 4 hours after treatment, treated mice sacrificed, and spleen were removed. As described above, ½ of each spleen was homogenized and plated to determine CFU/spleen. Intact tissue (remaining ½ spleen) was processed for fluorescence microscopy.

### Fluorescence microscopy

*host tissues* Spleens were harvested and immediately fixed in 4% PFA by incubating overnight at 4^o^ C. Tissues were frozen-embedded in Tissue Plus O.C.T. compound (Fisher Scientific) and cut by cryostat microtome into 10µm sections. Two sections were analyzed for each spleen. To visualize reporters, sections were thawed in PBS at room temperature, stained with Hoechst at a 1:10,000 dilution to detect host cell nuclei, washed in PBS, and coverslips were mounted using ProLong Gold (Life Technologies). Tissue was imaged as described above, with an Apotome.2 (Zeiss) for optical sectioning.

### Image analysis

Volocity image analysis software was used to quantify microcolony areas and fluorescence as previously described [3,20]. Two cross-sections were analyzed for each spleen, and every bacterial cell or clustered microcolony was imaged and analyzed within these sections. Spatial analyses were completed using Volocity software by sampling 4, ~ 4µm^2^ regions of interest (ROI) at both the centroid and periphery of each microcolony, and calculating the ratio of mCherry/GFP signal intensity in each ROI. Centroid measurements were captured in a cluster of 4 ROIs around the geometric centroid of each microcolony. Peripheral measurements were taken approximately every 90^o^ around each microcolony, sampling bacterial cells in contact with host cells. Each mCherry/GFP ratio was averaged to generate an average peripheral or centroid measurement for each microcolony, and these values were plotted. Individual fluorescent channel or spatial location values were also plotted using these ROIs. Fluorescent signals were quantified the same way from agarose droplets.

### Droplet generation

CAD-designed microfluidics chips with 38 independent devices controlled by two input ports were constructed according to published protocols [74,75] at the FabLab Micro and Nano fabrication laboratory at the University of Maryland, and devices were used as previously described [53]. Prior to droplet generation, each device was primed with HFE-7500 Novec oil (Oakwood Chemical) by connecting a 1ml syringe fitted with a 26-gauge needle to each input port via polyethylene (PE 20) tubing (BD). A third tube was inserted into the droplet collection port and waste was collected in a 1.5 ml microcentrifuge tube. After priming, the syringe connected to the oil-phase port was replaced with a syringe containing 1.5% FluoroSurfactant in HFE7500 Novec oil by removing the tubing from the needle of the priming syringe and placing the tubing onto the 1.5% FluoroSurfactant syringe. The syringe connected to the aqueous phase port was replaced with a 1 ml syringe containing bacteria diluted 1:2000 in 1% ultra-low-melt agarose, yielding approximately 5 x 10^6^ cells/ml. The oil-phase and aqueous-phase syringes were loaded onto separate programmable syringe pumps (Harvard Apparatus 11 Elite Series) set to 700 µl/hour and 200 µl/hour respectively. A heat lamp (VWR) was placed at a short distance (~ 0.5 m) from the syringe pump and tubing to prevent premature solidification of the agarose. Droplets were collected in a 1.5 ml microcentrifuge tube and incubated at 4° C for 30 minutes to solidify.

### Oil removal

Oil was removed from the droplets as previously described [53]*.* To remove oil from 200 µl of droplets, aliquots of 100 µl of droplets were moved into two separate 1.5 ml microcentrifuge tubes and 500 µl of 10% 1H, 1H, 2H, 2H-Perfluoro-1-octanol (PF) (Sigma) in Novec 7500 oil was added to each. The tubes were shaken vigorously and centrifuged for 30 seconds at 250 RCF. 400 µl PBS was added and the droplets were flicked into suspension then centrifuged for 30 seconds at 250 RCF. The PBS/droplet layers from each tube were combined in a new 1.5 ml microcentrifuge tube, centrifuged for 30 seconds at 250 RCF, and washed with 1 ml PBS. The remaining droplets were resuspended in 700 µl 2xYT broth.

### Magnesium oxalate droplet experiments

* *~ 400 µl of droplets were generated and separated as 200 µl aliquots resuspended in 1.5 ml microcentrifuge tubes containing 700 µl 2xYT. Both tubes were shaken gently then rotated at 37^o^ C. At 2 hours, MgCl_2_ (20mM) and sodium oxalate (20mM) were added to one tube while an equivalent amount of ddH_2_O (200µl) was added to the untreated tube. Samples were taken at 2-hour intervals by removing the tubes from the incubator, aliquoting 100 µl of each 2xYT-droplet mixture into new microcentrifuge tubes, centrifuging the tubes for 30 seconds at 250 RCF, and resuspending the droplets in 100 µl of 4% PFA.

## Supporting information

S1 Table Full RNA-seq dataset: list of genes with heightened reads in exponential phase. (XLS)

S2 Table Full RNA-seq dataset: list of genes with heightened reads in stationary phase. (XLS)

S3 Table Greene et al. raw datasets. All data included within the manuscript is provided in this Supporting Information file.
(XLSX)

S1 Fig *Yersinia pseudotuberculosis* ribosomal protein gene organization. Protein subunit names and gene names are shown. Arrows indicate promoter regions, direction indicates direction of transcription, determined using genomes NZ_CP009712.1 and NZ_CP032566.1. Vertical lines indicate genes are not adjacent. Horizontal black line indicates S17/*rpsQ* and L14/*rplN* are adjacent. Genes are color-coded based on fold increase (>5) in exponential phase cells. Created in BioRender. Davis, K. (2025) https://BioRender.com/36ckf9v.
(TIF)

S2 Fig S10 and T3SS reporter signals do not correlate in the absence of MgOx treatment. **A)** Correlation plot of fold change in single cell mCherry and GFP fluorescence from bacteria cultured in the absence (-) of MgOx. **(B)** Linear regression data indicating slope, R2, and significance of the lines of best fit shown in **(A).** Data represents 3 biological replicates for each strain and condition. Significantly non-zero slope indicates correlation between values.
(TIF)

S3 Fig MgOx treatment is not sufficient to alter S10 reporter activity. WT, *yopE::mCherry,* and *yopE::mCherry P*_*S10*_*::gfp-ssrA* strains were cultured at 26^o^ C in the presence (+) or absence (-) of MgOx for the indicated timepoints (hours, h). **(A)** Growth curve of strains with and without MgOx. Absorbance (A_600nm_) detected by plate reader at the indicated timepoints. Statistics compare the *yopE::mCherry P*_*S10*_*::gfp-ssrA* strain in the presence (+) or absence (-) of MgOx. Mean and standard deviation are shown. **(B)** Representative fluorescence microscopy images of bacteria from **(A)** immobilized on 1% agarose pads. Fold change in **(C)** mCherry (*yopE::mCherry*) reporter signal and **(D)** GFP (*P*_*S10*_*::gfp-ssrA*) reporter signal in the absence (-) or presence (+) of MgOx. Values quantified in individual bacteria by fluorescence microscopy. Single cell fluorescence was normalized to the average fluorescent value at 0h (value of 1, represented by dotted line). Each dot: individual cell, horizontal lines: median values. **(E)** Quantification of single cell bacterial areas (µm2) from samples in panels **(C)** and **(D)**. Horizontal lines: median values. All data represent 3 biological replicates for each strain and condition in this figure. Statistics: **(A)** Two-way ANOVA with Tukey’s multiple comparison test; **(C-E)** Kruskal Wallis one-way ANOVA with Dunn’s post-test; ****p < 0.0001, ***p < .001, **p < .01, ns: not-significant.
(TIF)

S4 Fig Quantification of single cell areas during MgOx treatment. WT *P*_*S10*_*::gfp-ssrA*, ∆lcrF *P*_*S10*_*::gfp-ssrA*, and ∆*iscR P*_*S10*_*::gfp-ssrA* were cultured at 37^o^ C in the presence (+) of MgOx. Single cell areas were quantified and raw values are shown (µm^2^). Statistics compare the WT strain to other strains at the same timepoint. Each dot: individual cell, horizontal lines: median values. All data represent 3 biological replicates for each strain and timepoint in this figure. Statistics: Kruskal Wallis one-way ANOVA with Dunn’s post-test; ****p < 0.0001, *p < .05, ns: not-significant.
(TIF)

S5 Fig WT and ∆*lcrF* strains grew similarly in M9 media and exhibit similar gentamicin susceptibility. WT and ∆*lcrF* strains were grown in M9 media [57] and bacterial numbers were quantified by plating for CFUs. **(A)** T3SS expression was detected by plate reader (560ex/610em) at the indicated timepoints using a *yopE::mCherry* reporter strain grown in parallel (red lines). Dotted horizontal line: value of 1, represents either fluorescence at time 0h, or change in growth relative to the 2h (hatched black lines) or 3h (solid black lines) start of plating. 2h and 3h were chosen to assess growth and antibiotic susceptibility at different points in exponential phase. **(B)** Gentamicin (10µg/ml) was added to aliquots of cultures at the indicated timepoints and bacterial survival was assessed based on quantifying CFUs/ml. Fold change in CFUs is shown relative to start of treatment (dotted line at value of 1). One replicate is shown for the 2h experiment (hatched black lines), 3 biological replicates are shown for the 3h experiment (solid black lines). Statistics: Two-way ANOVA with Tukey’s multiple comparison test, statistics shown represent comparisons between WT and ∆*lcrF*; ns: not-significant.
(TIF)
