# Supplementary figures and images for: Yersinia pseudotuberculosis growth arrest during type-III secretion system expression is associated with altered ribosomal protein expression and decreased gentamicin susceptibility
Source: PLoS Pathog. 2025 Jul 7;21(7):e1012548. doi: 10.1371/journal.ppat.1012548 (PMC12244630; doi:10.1371/journal.ppat.1012548)

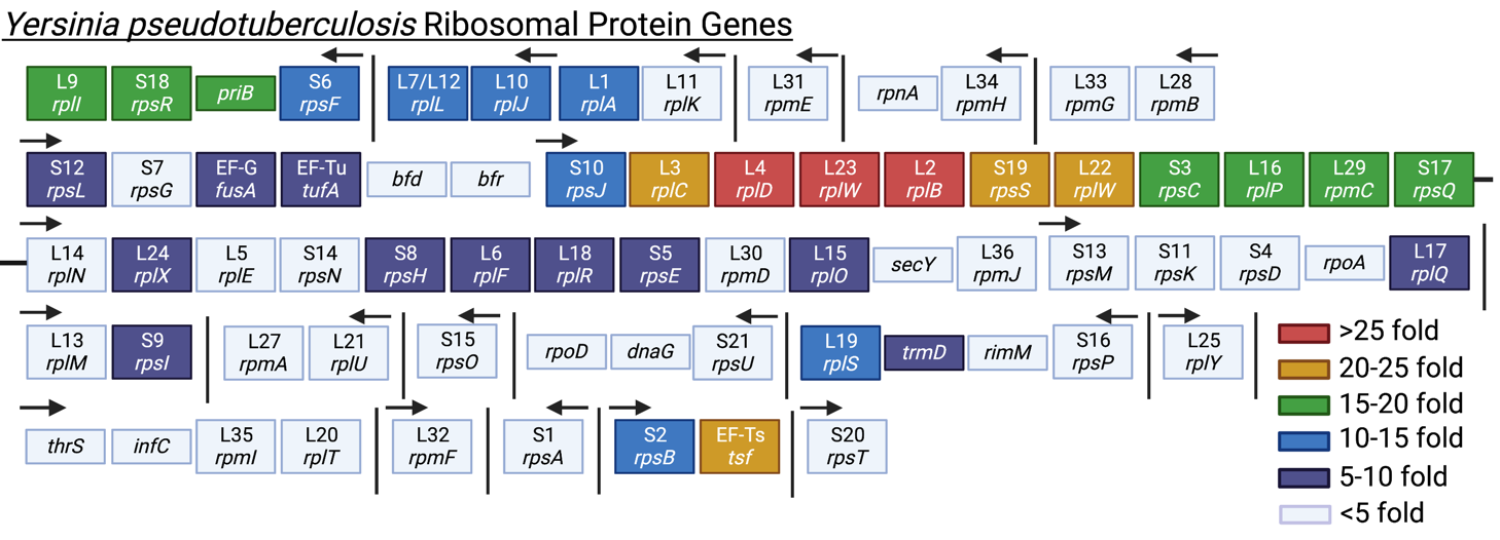

Supplement: S1 Fig — Protein subunit names and gene names are shown. Arrows indicate promoter regions, direction indicates direction of transcription, determined using genomes NZ_CP009712.1 and NZ_CP032566.1. Vertical lines indicate genes are not adjacent. Horizontal black line indicates S17/rpsQ and L14/rplN are adjacent. Genes are color-coded based on fold increase (>5) in exponential phase cells. Created in BioRender. Davis, K. (2025) https://BioRender.com/36ckf9v. (TIF) [file ppat.1012548.s004.tif]

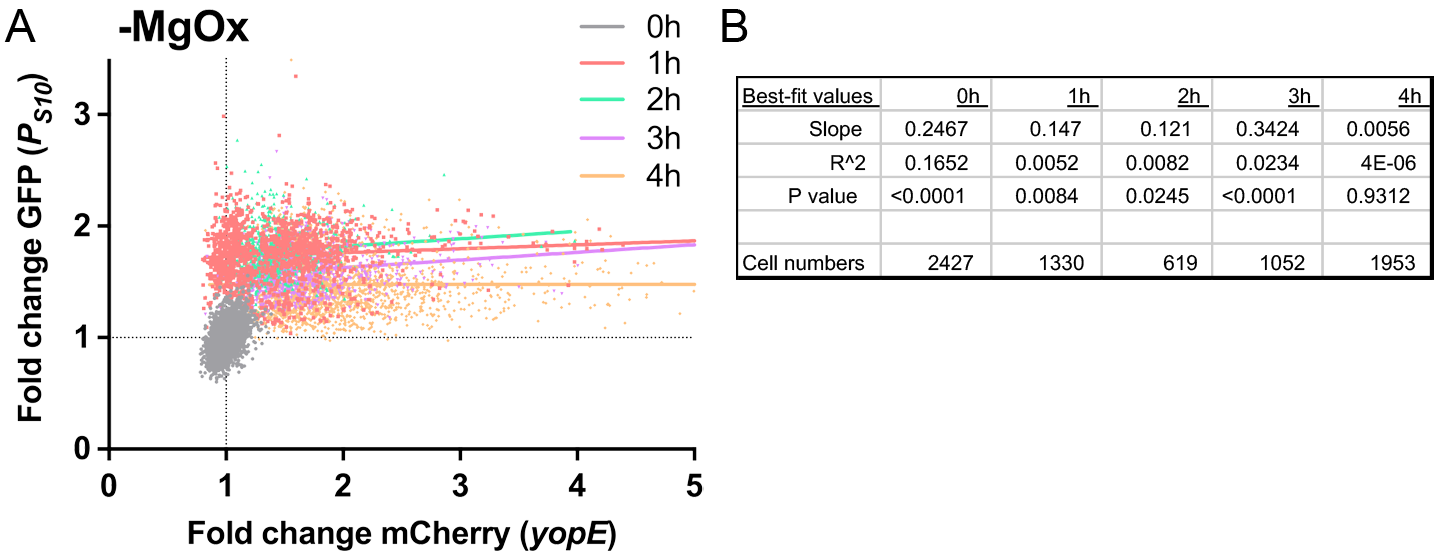

Supplement: S2 Fig — A) Correlation plot of fold change in single cell mCherry and GFP fluorescence from bacteria cultured in the absence (-) of MgOx. (B) Linear regression data indicating slope, R2, and significance of the lines of best fit shown in (A). Data represents 3 biological replicates for each strain and condition. Significantly non-zero slope indicates correlation between values. (TIF) [file ppat.1012548.s005.tif]

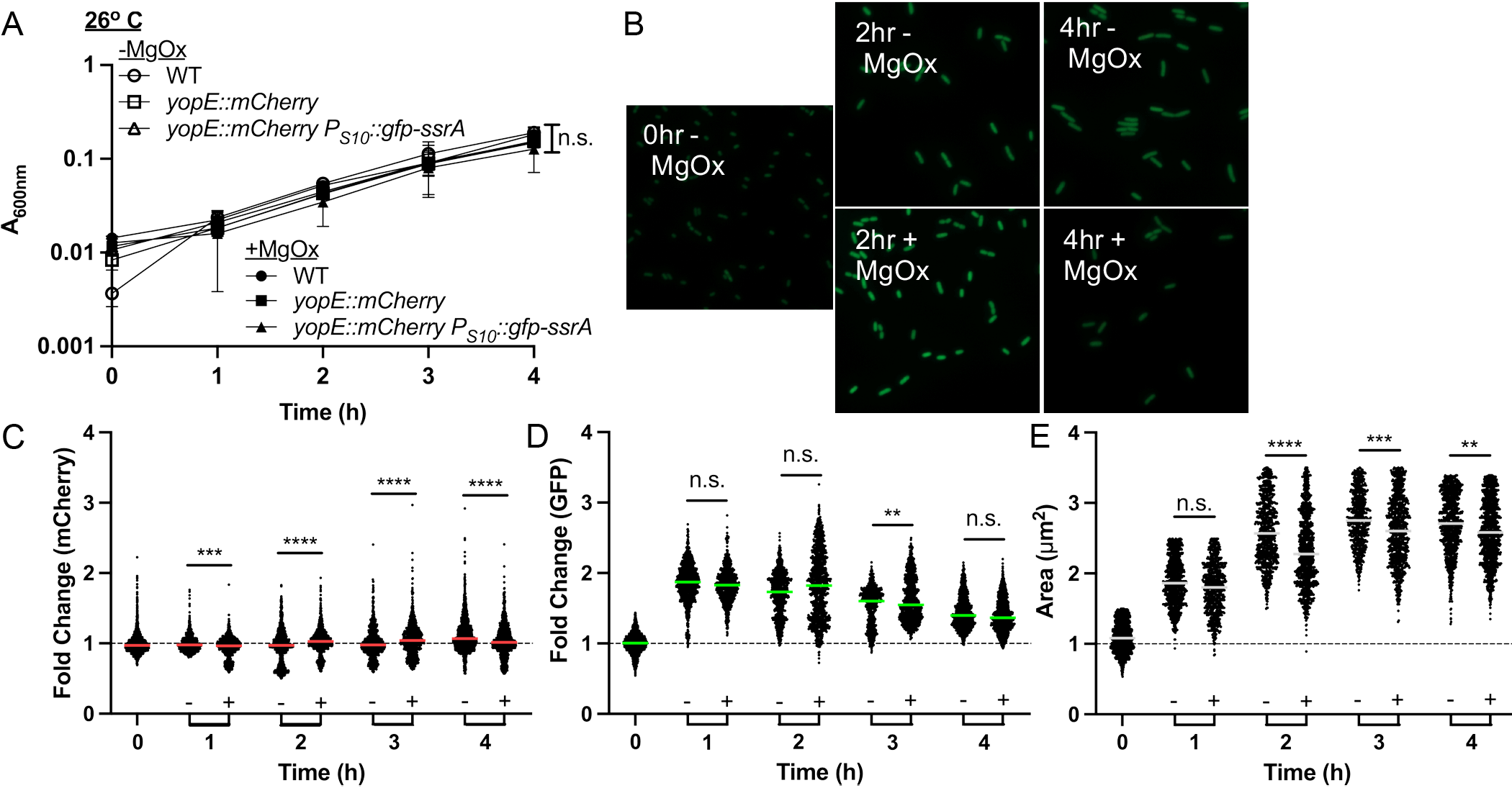

Supplement: S3 Fig — WT, yopE::mCherry, and yopE::mCherry PS10::gfp-ssrA strains were cultured at 26o C in the presence (+) or absence (-) of MgOx for the indicated timepoints (hours, h). (A) Growth curve of strains with and without MgOx. Absorbance (A600nm) detected by plate reader at the indicated timepoints. Statistics compare the yopE::mCherry PS10::gfp-ssrA strain in the presence (+) or absence (-) of MgOx. Mean and standard deviation are shown. (B) Representative fluorescence microscopy images of bacteria from (A) immobilized on 1% agarose pads. Fold change in (C) mCherry (yopE::mCherry) reporter signal and (D) GFP (PS10::gfp-ssrA) reporter signal in the absence (-) or presence (+) of MgOx. Values quantified in individual bacteria by fluorescence microscopy. Single cell fluorescence was normalized to the average fluorescent value at 0h (value of 1, represented by dotted line). Each dot: individual cell, horizontal lines: median values. (E) Quantification of single cell bacterial areas (µm2) from samples in panels (C) and (D). Horizontal lines: median values. All data represent 3 biological replicates for each strain and condition in this figure. Statistics: (A) Two-way ANOVA with Tukey’s multiple comparison test; (C-E) Kruskal Wallis one-way ANOVA with Dunn’s post-test; ****p < 0.0001, ***p < .001, **p < .01, ns: not-significant. (TIF) [file ppat.1012548.s006.tif]

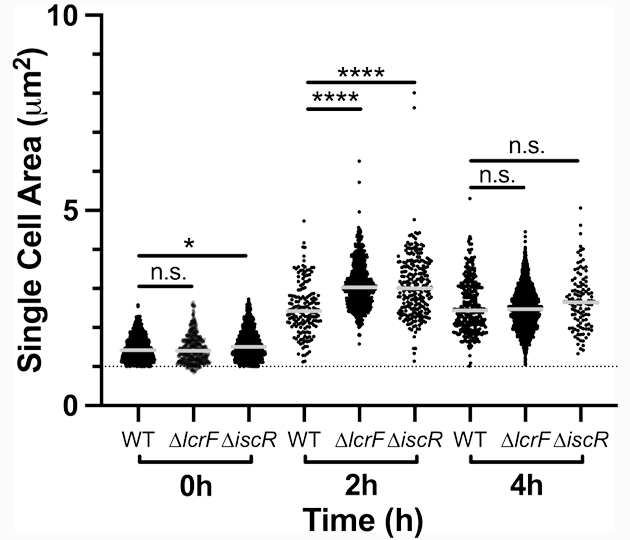

Supplement: S4 Fig — WT PS10::gfp-ssrA, ∆lcrF PS10::gfp-ssrA, and ∆iscR PS10::gfp-ssrA were cultured at 37o C in the presence (+) of MgOx. Single cell areas were quantified and raw values are shown (µm2). Statistics compare the WT strain to other strains at the same timepoint. Each dot: individual cell, horizontal lines: median values. All data represent 3 biological replicates for each strain and timepoint in this figure. Statistics: Kruskal Wallis one-way ANOVA with Dunn’s post-test; ****p < 0.0001, *p < .05, ns: not-significant. (TIF) [file ppat.1012548.s007.tif]

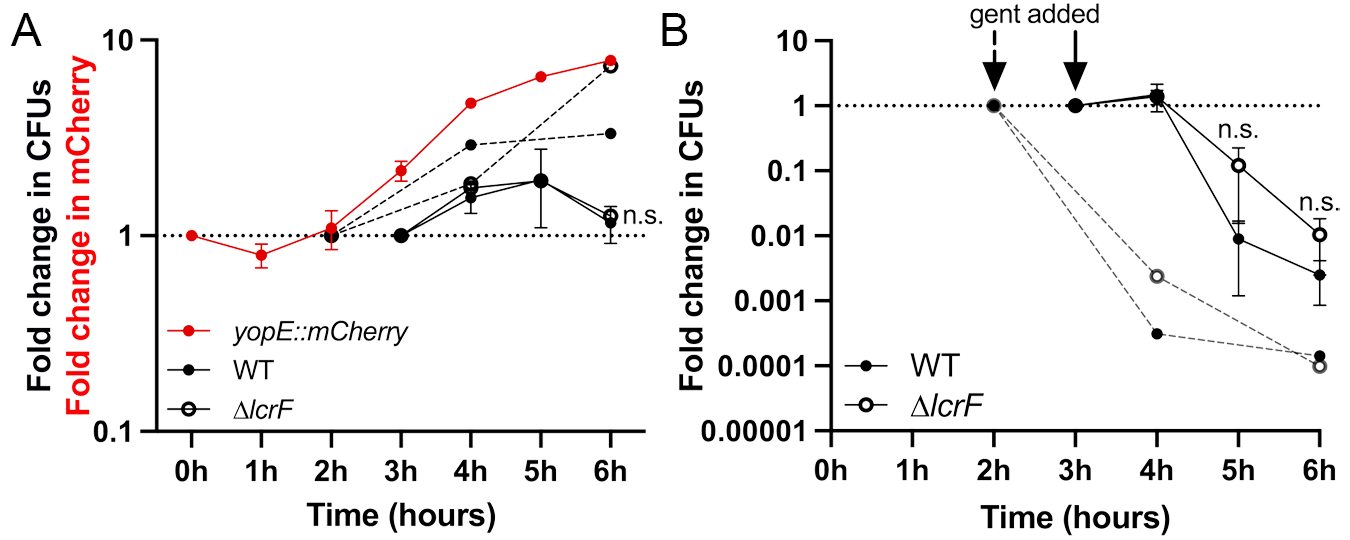

Supplement: S5 Fig — WT and ∆lcrF strains were grown in M9 media [57] and bacterial numbers were quantified by plating for CFUs. (A) T3SS expression was detected by plate reader (560ex/610em) at the indicated timepoints using a yopE::mCherry reporter strain grown in parallel (red lines). Dotted horizontal line: value of 1, represents either fluorescence at time 0h, or change in growth relative to the 2h (hatched black lines) or 3h (solid black lines) start of plating. 2h and 3h were chosen to assess growth and antibiotic susceptibility at different points in exponential phase. (B) Gentamicin (10µg/ml) was added to aliquots of cultures at the indicated timepoints and bacterial survival was assessed based on quantifying CFUs/ml. Fold change in CFUs is shown relative to start of treatment (dotted line at value of 1). One replicate is shown for the 2h experiment (hatched black lines), 3 biological replicates are shown for the 3h experiment (solid black lines). Statistics: Two-way ANOVA with Tukey’s multiple comparison test, statistics shown represent comparisons between WT and ∆lcrF; ns: not-significant. (TIF) [file ppat.1012548.s008.tif]
